# Supplementary material for: Identification of a Small Molecule Inhibitor of Hyaluronan Synthesis, DDIT, Targeting Breast Cancer Cells
Source: Cancers (Basel). 2022 Nov 25;14(23):5800. doi: 10.3390/cancers14235800 (PMC9741431; doi:10.3390/cancers14235800)
Supplement: Supplementary file 1 [file cancers-14-05800-s001.zip › cancers-1991078-SI.pdf]

Supplementary Material

Table S1: Structure and nomenclature of hyaluronan synthase inhibitors

Compound	Structure	Name	Classification
**1**	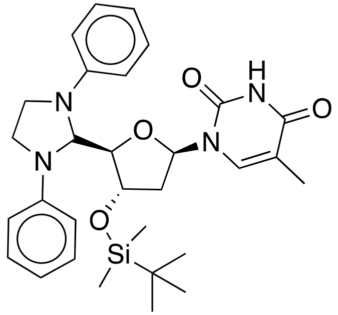	**3’-O-(TBDMS)-5’-Deoxy-5’-(1,3-Diphenyl-2-Imidazolidinyl)- Thymidine**	**Thymidine Analog**
**2**	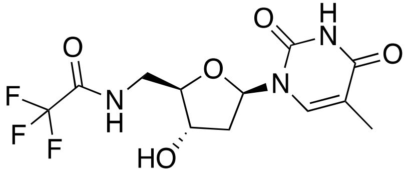	**5’-Trifluoroacetamido-5’-Deoxythymidine**	**Thymidine Analog**
**3**	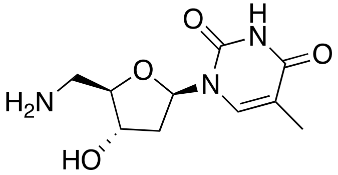	**5’-Amino-5’-Deoxythymidine**	**Thymidine Analog**

**4**	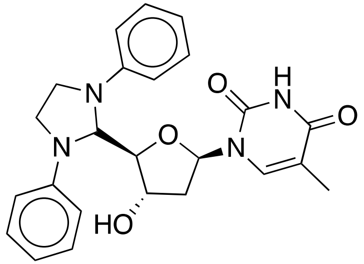	**5'-Deoxy-5'-(1,3-Diphenyl-2-Imidazolidinyl)-Thymidine**<br><br>**DDIT**	**Thymidine Analog**
**5**	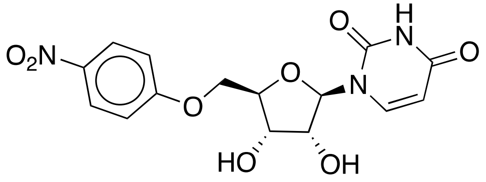	**5'-O-(4-Nitrophenyl)-Uridine**	**Uridine Analog**
**6**	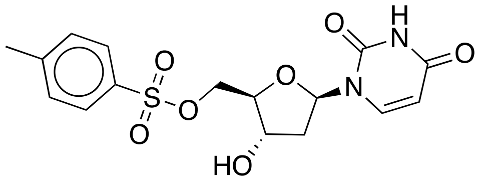	**2'-Deoxy-5'-O-p-Toluenesulfonyluridine**	**Uridine Analog**
**7**	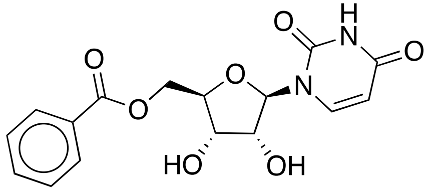	**5'-O-Benzoyluridine**	**Uridine Analog**
**8**	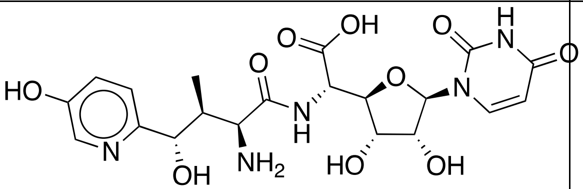	**Nikkomycin Z**	**Natural Product**<br>**(Uridine Analog)**

**9**	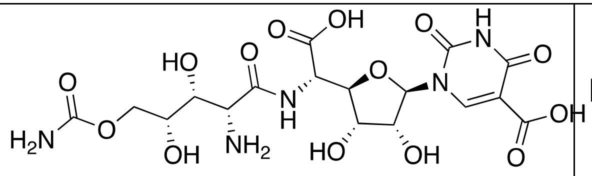	**Polyoxin D**	**Natural Product (Uridine Analog)**
**10**	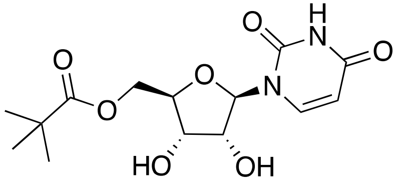	**5’-O-Pivaloyluridine**	**Uridine Analog**
**11**	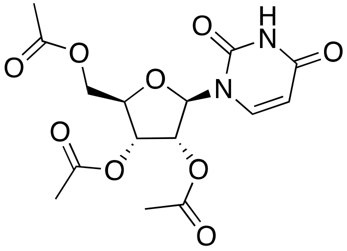	**Uridine Triacetate**	**Uridine Analog**
**12**	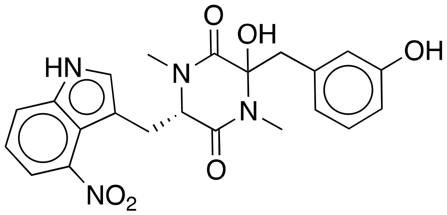	**Thaxtomin A**	**Cellulose Synthesis Inhibitor**
**13**	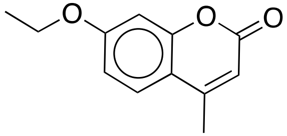	**Morlin**	**Cellulose Synthesis Inhibitor**

Table S2: Vendor information of the compounds used in the present study.

Compound	IUPAC Name	Vendor	CAS#
1	1-((2R,4S,5R)-4-((tert-butyldimethylsilyl)oxy)-5-(1,3-diphenylimidazolidin-2-yl)tetrahydrofuran-2-yl)-5-methylpyrimidine-2,4(1H,3H)-dione	BERRY & ASSOCIATES	149741-58-2
2	2,2,2-trifluoro-N-(((2R,3S,5R)-3-hydroxy-5-(5-methyl-2,4-dioxo-3,4-dihydropyrimidin-1(2H)-yl)tetrahydrofuran-2-yl)methyl)acetamide	BERRY & ASSOCIATES	55812-00-5
3	1-((2R,4S,5R)-5-(aminomethyl)-4-hydroxytetrahydrofuran-2-yl)-5-methylpyrimidine-2,4(1H,3H)-dione	BERRY & ASSOCIATES	25152-20-9
4	1-((2R,4S,5R)-5-(1,3-diphenylimidazolidin-2-yl)-4-hydroxytetrahydrofuran-2-yl)-5-methylpyrimidine-2,4(1H,3H)-dione	BERRY & ASSOCIATES	869355-30-6
5	1-((2R,3R,4S,5R)-3,4-dihydroxy-5-((4-nitrophenoxy)methyl)tetrahydrofuran-2-yl)pyrimidine-2,4(1H,3H)-dione	AKOS	39946-91-3
6	((2R,3S,5R)-5-(2,4-dioxo-3,4-dihydropyrimidin-1(2H)-yl)-3-hydroxytetrahydrofuran-2-yl)methyl 4-methylbenzenesulfonate	CARBOSYNTH	27999-47-9
7	((2R,3S,4R,5R)-5-(2,4-dioxo-3,4-dihydropyrimidin-1(2H)-yl)-3,4-	LIFE CHEMICALS	54618-06-3

	dihydroxytetrahydrofuran-2-yl)methyl benzoate		
8	(S)-2-((2S,3S,4S)-2-amino-4-hydroxy-4-(5-hydroxypyridin-2-yl)-3-methylbutanamido)-2-((2R,3S,4R,5R)-5-(2,4-dioxo-3,4-dihydropyrimidin-1(2H)-yl)-3,4-dihydroxytetrahydrofuran-2-yl)acetic acid	SIGMA	59456-70-1
9	1-((2R,3R,4S,5R)-5-((S)-((2R,3R,4R)-2-amino-5-(carbamoyloxy)-3,4-dihydroxypentanamido)(carboxy)methyl)-3,4-dihydroxytetrahydrofuran-2-yl)-2,4-dioxo-1,2,3,4-tetrahydropyrimidine-5-carboxylic acid	EMD CHEMICALS	22976-86-9
10	((2R,3S,4R,5R)-5-(2,4-dioxo-3,4-dihydropyrimidin-1(2H)-yl)-3,4-dihydroxytetrahydrofuran-2-yl)methyl pivalate	AMBINTER	6554-06-9
11	(2R,3R,4R,5R)-2-(acetoxymethyl)-5-(2,4-dioxo-3,4-dihydropyrimidin-1(2H)-yl)tetrahydrofuran-3,4-diyl diacetate	ARK PHARM	4105-38-8
12	(6S)-3-hydroxy-3-(3-hydroxybenzyl)-1,4-dimethyl-6-((4-nitro-1H-indol-3-yl)methyl)piperazine-2,5-dione	SANTA CRUZ	122380-18-1
13	7-ethoxy-4-methyl-2H-chromen-2-one	SIGMA	87-05-8

Table S3: RT-qPCR primer sequences used in the present study.

Target gene	Primer Sequence (5'-3')	$T_{Annealing}$
CD44s	Sense: ATAATAAAGGAGCAGCACTTCAGGA<br>Anti-sense: ATAATTTGTGTCTTGGTCTCTGGTAGC	60°C
CD44v3	Sense: ATAATGGCTGGGAGCCAAATGAAGAAA<br>Anti-sense: ATAATCATCATCATCAATGCCTGATCCAGA	60°C
CD44v6	Sense: ATAATCAGAAGGAACAGTGGTTTGGCA<br>Anti-sense: ATAATGTCTTCTTTGGGTGTTTGGCGA	60°C
CD44v9	Sense: ATAATGAGCTTCTCTACATCACATGAAGGC<br>Anti-sense: TAATGTCAGAGTAGAAGTTGTTGGATGGTC	60°C
RHAMM	Sense: GCAAACACTGGATGAGCTTGA<br>Anti-sense: TGGTCTGCAGATCTAGAAGCA	
HAS1	Sense: GGAATAACCTCTTGCAGCAGTTTC<br>Anti-sense: GCCGGTCATCCCCAAAAG	60°C
HAS2	Sense: TCGCAACACGTAACGCAAT<br>Anti-sense: ACTTCTCTTTTTCCACCCCATTT	60°C
HAS3	Sense: AACAAGTACGACTCATGGATTTCCT<br>Anti-sense: GCCCGCTCCACGTTGA	60°C
HYAL-1	Sense: GATTGCAGTGTCTTCGATGTGGTA<br>Anti-sense: GGGAGCTATAGAAAATTGTCATGTCA	60°C
HYAL-2	Sense: CTAATGAGGGTTTTGTGAACCAGAATAT<br>Anti-sense: GCAGAATCGAAGCGTGGATAC	60°C
TMEM2	Sense: GGAATAGGACTGACCTTTGCCAG<br>Anti-sense: TTCTGACCACCCTGAAAGCCGT	60°C
KIAA1199/CEMIP	Sense: ACCGAGCACATTCCAACTACCG<br>Anti-sense: GGCAGAGATGATTGAGAGGAACG	60°C

OCT4	Sense: AGTGCCCGAAACCCACACT<br>Anti-sense: CTTCTGGCGCCGGTTACA	60°C
NANOG	Sense: TGCCTCACACGGAGACTGTCT<br>Anti-sense: AGTGGGTTGTTTGCCTTTGG	60°C
SOX2	Sense: ACACCCTGATCTGGCATGGA<br>Anti-sense: GGCTGTTGCCTGGCTTCTC	60°C
TBP	Sense: TGGCGTGTGAAGATAACCCAA<br>Anti-sense: TCTTGGCAAACCAGAAACCCT	60°C

Table S4: Antibodies used in the present study.

Antigen	Specie	Catalog No.	Dilution
CD44	Mouse	Hermes-3, kindly provided by Dr. Jalkanen	1 µg/mL
HAS2	Mouse	Santa Cruz Biotechnology, Inc, A7, #sc-514737	1:200
c-myc	Mouse	Santa Cruz Biotechnology, Inc, 9E10, #sc-40	1:200
Cyclin B1	Rabbit	Cell Signaling, #4138	1:1,000
Cyclin E1	Mouse	Cell Signaling, #4129	1:1,000
Cyclin D1	Mouse	Santa Cruz Biotechnology, Inc, A-12, #sc-8396	1:200
Caspase-3	Rabbit	Cell Signaling, #9665	1:1,000
phospho-S6 (Ser235/236)	Rabbit	Cell Signaling, #2211	1:1,000
S6	Mouse	Cell Signaling, #2317	1:1,000
p27	Rabbit	Cell Signaling, #3686	1:1,000
GAPDH	Rabbit	Cell Signaling, 14C10, #2118	1:2,000
Anti-mouse IgG (H+L) Secondary antibody, HRP	Goat	ThermoFischer Scientific, #31430	1:10,000
Anti-rabbit IgG (H+L) Secondary antibody, HRP	Goat	ThermoFischer Scientific, #31460	1:10,000

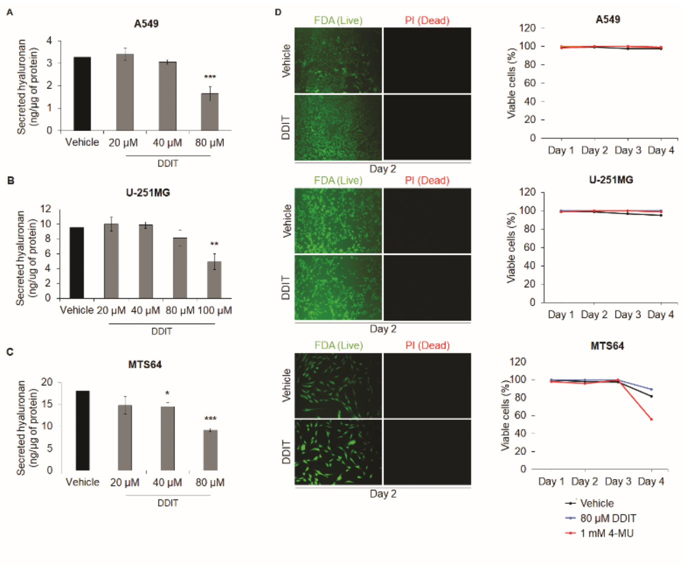

Figure S1: Dose-response analysis of the effect of DDIT on hyaluronan synthesis and toxicity. Hyaluronan secreted by A549 lung adenocarcinoma cells (A), U-251MG glioblastoma cells (B) and MTS64 normal dermal fibroblasts (C) after treatment with vehicle (0.04% DMSO) or DDIT (20, 40, 80 and 100 μM) in serum-free medium, for 24 hours. (E) Viability of A549 lung adenocarcinoma cells, U-251MG glioblastoma cells and MTS64 normal dermal fibroblasts was determined by FDA/PI staining of live (green) or dead (red) cells, after treatment with vehicle (0.04% DMSO), or DDIT (80 μM) in serum-free medium, for 1, 2, 3 and 4 days. Images show representative staining of the respective cell lines from day two. The images were captured with a 5x objective.

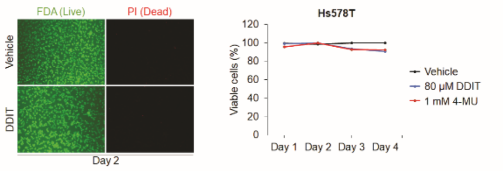

Figure S2: Viability of Hs578T cells. The viability of the cells was determined by FDA/PI staining of live (green) or dead (red) cells, after treatment with 100 μM DDIT .

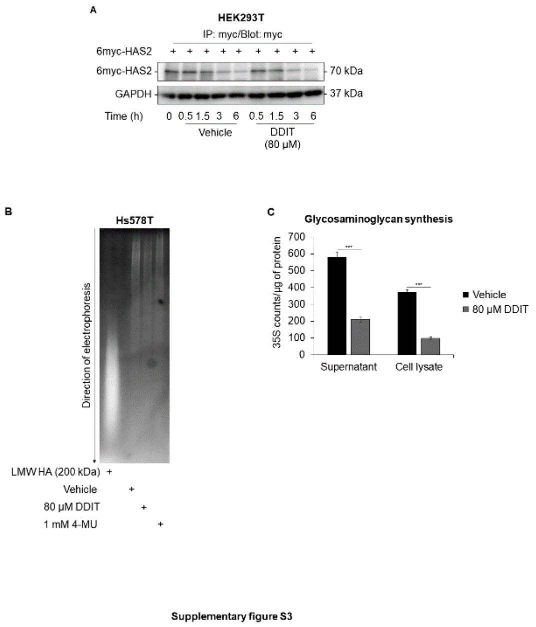

Figure S3: Effect of DDIT on 6myc-HAS2 stability, size of hyaluronan, and glycosaminoglycan synthesis. HEK293T cells were transfected with 6myc-tagged HAS2 for 24 hours. Then, lysates from untreated or treated cells with 20 µM cycloheximide (for different time points) were subjected to immunoprecipitation followed by protein separation on SDS-PAGE (A). The size of hyaluronan in 24 h conditioned media of untreated or DDIT- and 4-MU-treated cultures was analyzed by agarose gel electrophoresis (B). Ion-exchange chromatography of Hs578T cells treated or not with DDIT as described in Materials and Methods.

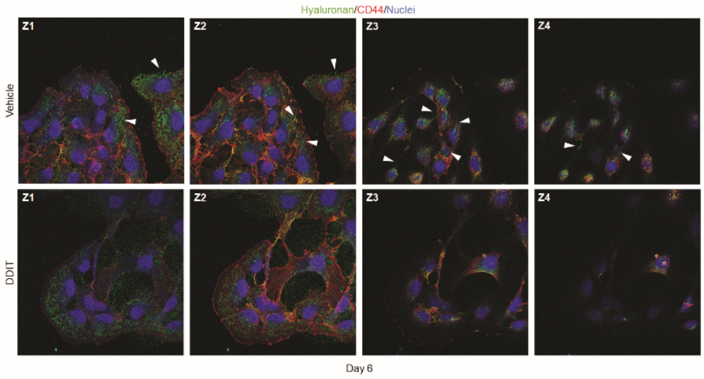

Figure S4: DDIT inhibits formation of hyaluronan cable-like structures. Z-stacks of confocal imaging of Hs578T breast cancer cells stained for hyaluronan (green) and CD44 (red) after treatment with vehicle (0.04% DMSO) or DDIT (80 µM) in 10% FBS, for 6 days. Arrow heads indicates hyaluronan cable-like structures.

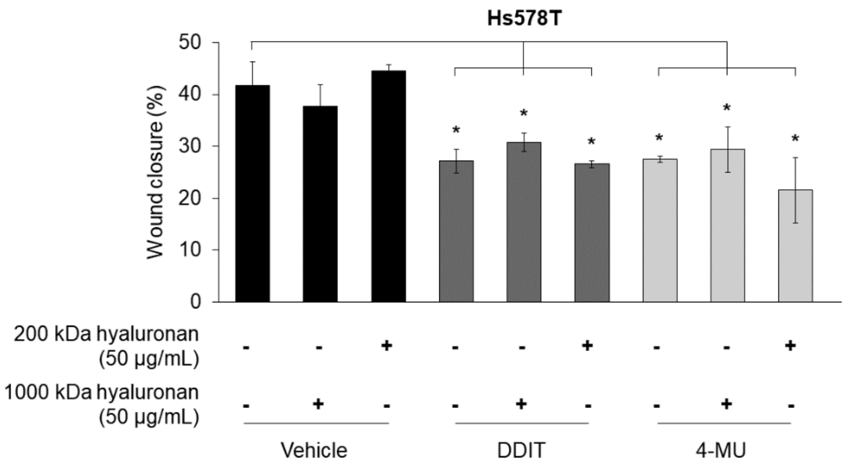

Figure S5: Exogenous hyaluronan does not recover breast cancer cell migration after hyaluronan synthesis inhibition. Wound healing of Hs578T breast cancer cells after treatment with vehicle (0.04% DMSO), DDIT (80 µM), 4-MU (1 mM) and 1000 or 200 kDa exogenous hyaluronan (50 µg/mL) in 10% FBS, for 12 hours.

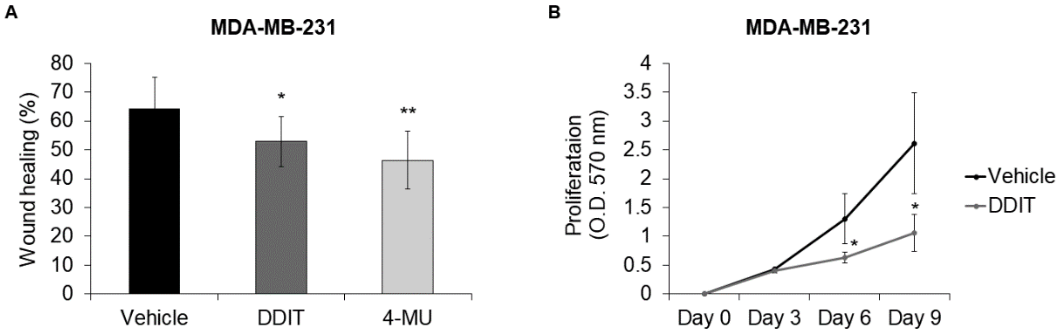

Figure S6: DDIT inhibits migration and proliferation of MDA-MB-231. (A) Wound healing of MDA-MB-231 breast cancer cells after treatment with vehicle (0.04% DMSO), DDIT (80 µM) or 4-MU (1 mM) in medium containing 10% FBS for 24 hours. (B) Proliferation of MDA-MB-231 breast cancer cells after treatment with vehicle (0.04% DMSO) or DDIT (80 µM) for 3, 6 and 9 days, in medium containing 10% FBS.

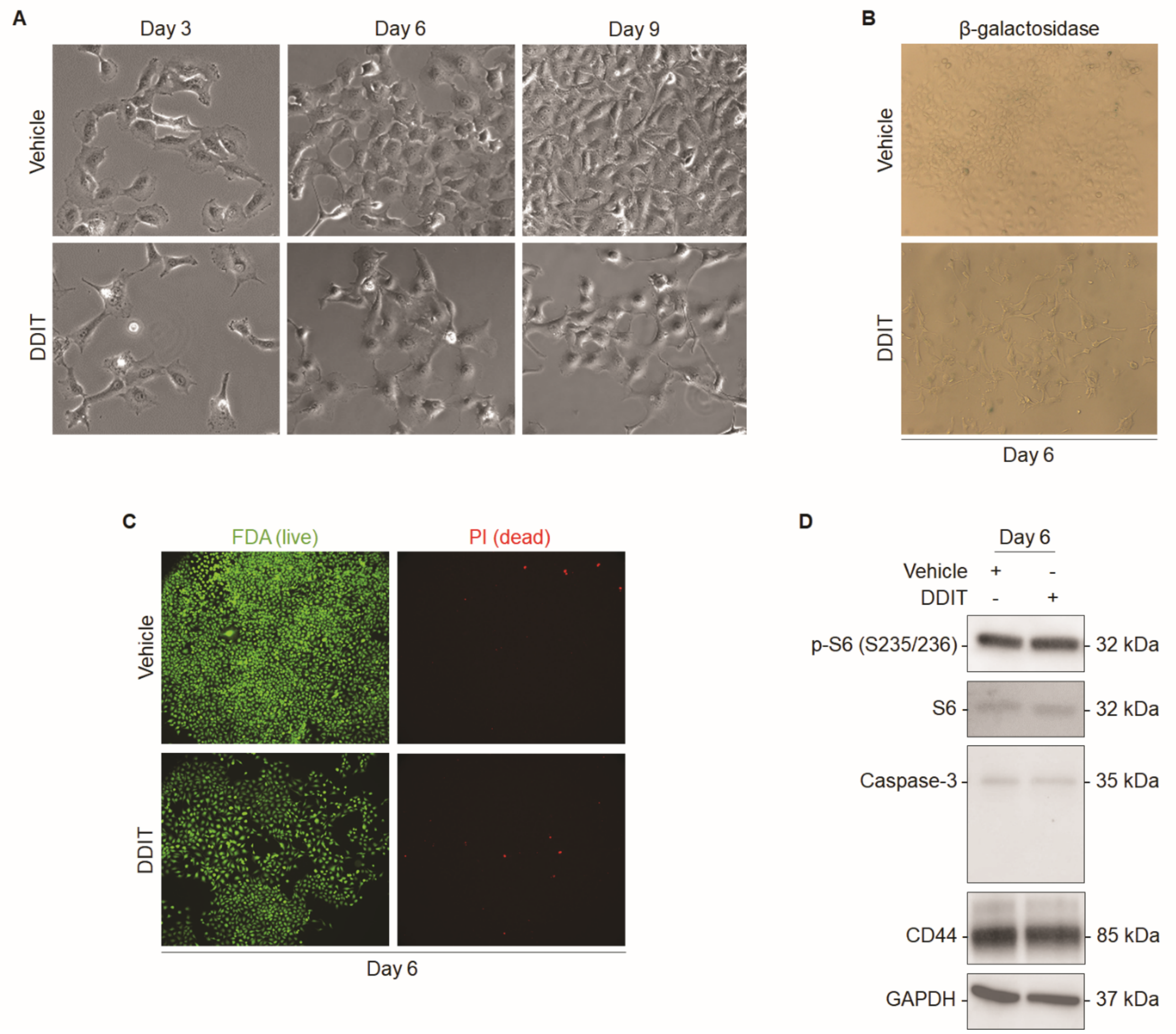

Figure S7: Long-term treatment with DDIT does not induce senescence or apoptosis in breast cancer cells. (A) Phase contrast images of Hs578T cells after 3, 6 and 9 days of treatment with vehicle (0.04% DMSO) or DDIT (80 μM) in medium containing 10% FBS and high glucose concentration. The images were captured with a 10X objective. (B-C) β-galactosidase (B) and FDA/PI (C) staining of live (green) or dead (red) Hs578T cells after 6 days of treatment with vehicle or DDIT in 10% FBS high glucose medium. The images were captured with a 5x objective. (D) Immunoblotting for p-S6 (S235/236)/S6, caspase-3, CD44 and GAPDH in total cell lysates of Hs578T after treatment with vehicle or DDIT for 6 days, in medium supplemented with 10% FBS.

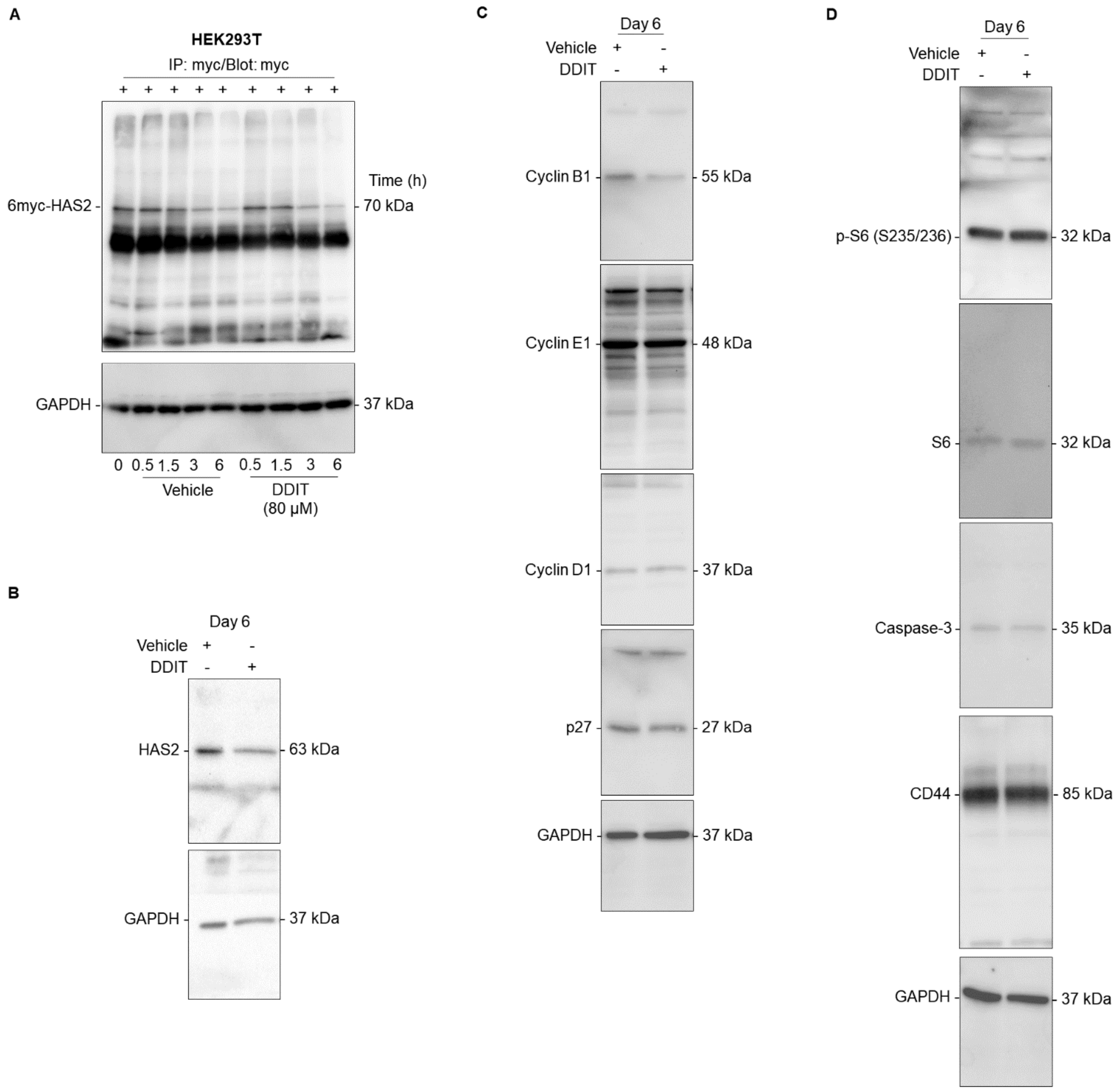

Figure S8: Uncropped blots corresponding to (A) Fig. S3A, (B) Fig. 8C, (C) Fig. 8E and (D) Fig. S7D.
